# Administration of Cimetidine for Calcific Tendinitis of the Rectus Femoris: Five Cases

**DOI:** 10.7759/cureus.61002

**Published:** 2024-05-24

**Authors:** Ryo Mitsutake, Masayuki Takakuwa, Hiromasa Tanino, Hiroshi Ito

**Affiliations:** 1 Department of Orthopaedic Surgery, Asahikawa Medical University, Asahikawa, JPN; 2 Department of Orthopaedic Surgery, Takakuwa Orthopaedic Nagayama Clinic, Asahikawa, JPN

**Keywords:** hip pain, acute, cimetidine, rectus femoris, calcific tendinitis

## Abstract

Calcific tendinitis of the rectus femoris is rare. This clinical report presents five cases of management of calcific tendinitis of the rectus femoris. Between July 2018 and March 2023, five patients visited our institution, where they were treated for calcific tendinitis of the rectus femoris. All patients presented with severe acute hip pain. Radiographs, computed tomography, magnetic resonance imaging, and an ultrasound examination of the hip showed calcification outside the joint, suggesting calcific tendinitis of the rectus femoris. All patients were orally administered 200 mg cimetidine and nonsteroidal anti-inflammatory drugs twice daily. A pain-free status was achieved in 2 weeks on average. Calcium deposits disappeared in three patients and decreased in two. Symptoms did not recur. Furthermore, no recurrence or enlargements in calcium deposits were observed. It appears to be an effective treatment for calcific tendinitis of the rectus femoris; however, the underlying mechanisms of action of cimetidine on calcific tendinitis have not yet been elucidated in detail.

## Introduction

Painful periarticular calcification commonly occurs within the rotator cuff of the shoulder [1], and sometimes at the wrist, elbow, knee, and neck [2,3]. Calcific tendinitis of the hip is relatively rare and was first recognized in 1967 by King and Vanderpool [4]. Since then, only 23 cases have been reported, as summarized by Kobayashi et al. [5]. Calcific tendinitis is an acute inflammatory reaction and a type of calcium hydroxyapatite crystal deposition disease. Pain or tenderness around the affected area can be so extreme that the range of motion is severely limited. A previous study reported that out of 12 histologically verified cases of calcific tendinitis adjacent to joints other than the shoulder, the preoperative clinical diagnosis was accurate in only one patient [6]. Unfamiliarity with calcific tendinitis in joints other than the shoulder can lead to misdiagnosis resulting in inappropriate treatment and delayed recovery [7]. When present in atypical locations, it is often misdiagnosed as an infection or a neoplasm. Standard treatments for calcific tendinitis include nonsteroidal anti-inflammatory drugs (NSAIDs), local steroids, and anesthetic injections [8]. Needle aspiration, shock wave therapy, and surgery need to be considered when conservative treatment fails. While the pathophysiologic factors remain under debate, a previous study indicated that cimetidine showed effectiveness in treating calcific tendinitis of the elbow or shoulder in uncontrolled studies [9,10]. However, the mechanisms of the action of cimetidine on calcific tendinitis have not been elucidated in detail, and its effectiveness as a treatment for calcific tendinitis of the hip has not yet been demonstrated. Five cases of calcific tendinitis of the rectus femoris treated using cimetidine were described herein.

## Case presentation

Between July 2018 and March 2023, 5 patients visited our institution, where they were treated for calcific tendinitis of the rectus femoris and subsequently followed up for at least 1 year: 5 hips in 5 patients (one male and four females) with the acute onset of pain in the hip had radiographically verified calcific tendinitis. The mean age of patients was 48.4 years (range: 22-76 years) and none had a previous history of hip conditions or any injury of note. The affected hips were held at 20° to 30° of flexion and all movements were limited by pain. All patients exhibited limping on the affected side. In all cases, radiographs and computed tomography (CT) of the hip showed an area of calcification near the superior lip of the acetabulum or adjacent to the anterior inferior iliac spine. In all cases, magnetic resonance imaging (MRI) and ultrasound examinations showed calcification within the rectus femoris and excluded intra-articular lesions. MRI and ultrasound examinations revealed inflammatory changes in the surrounding soft tissues. The pretreatment diagnosis was calcific tendinitis of the rectus femoris.

Oral NSAIDs and 200 mg cimetidine twice daily were administered. Other treatments, such as needle aspiration, local steroid injections, shock wave therapy, and surgery, were avoided in this case series. No patients in this case series were taking other anti-reflux or anti-esophagitis medications at the time of presentation. We continued to prescribe cimetidine for 1 month and assessed changes in calcification on radiographs at 1 month. We continued to prescribe NSAIDs until patients achieved a pain-free status. Anteroposterior radiographs and CT were acquired, and the sizes of calcium deposits were graded as follows: “disappearance”, “reduction”, or “no change”, compared to before treatment. The sizes of the calcium deposits were calculated on an anteroposterior radiograph. The patients were interviewed about their peak pain. We assessed visual analogue scale (VAS) for pain. VAS for pain was measured on a 100-point scale, with 0 indicating favorable results (i.e., no pain) and 100 indicating unfavorable results (i.e., unbearable pain).

The details of 5 patients with calcific tendinitis of rectus femoris are summarized in Table 1. 

**Table 1:** Details of five patients with calcific tendinitis of rectus femoris VAS: visual analog scale

	Case 1	Case 2	Case 3	Case 4	Case 5
Age (years)	52	76	48	22	44
Gender	Female	Male	Female	Female	Female
Affected side	Left	Right	Right	Left	Left
Size before cimetidine treatment (mm^2^)	24	55	28	40	21
Duration of symptom (days)	4	3	2	4	2
Peak pain (VAS)	70	82	68	78	76
Time of pain-free status (weeks)	2	2	4	1	1
Change in calcium deposit	Reduction	Disappearance	Reduction	Disappearance	Disappearance
Size after cimetidine treatment (mm^2^)	12	0	15	0	0

After initiation of cimetidine, all patients obtained relief from pain within 3 days. A pain-free status was achieved in 2.0 weeks on average (range: 1-4 weeks). 1 month after initiation of cimetidine treatment, X-rays showed the ‘‘disappearance’’ of calcifications in 3 out of 5 patients and the ‘‘reduction’’ in the remaining 2. Symptoms did not recur during the follow-up period. No recurrence or enlargements in calcium deposits were observed. There were no side effects with the treatment of cimetidine.

**Figure 1:**
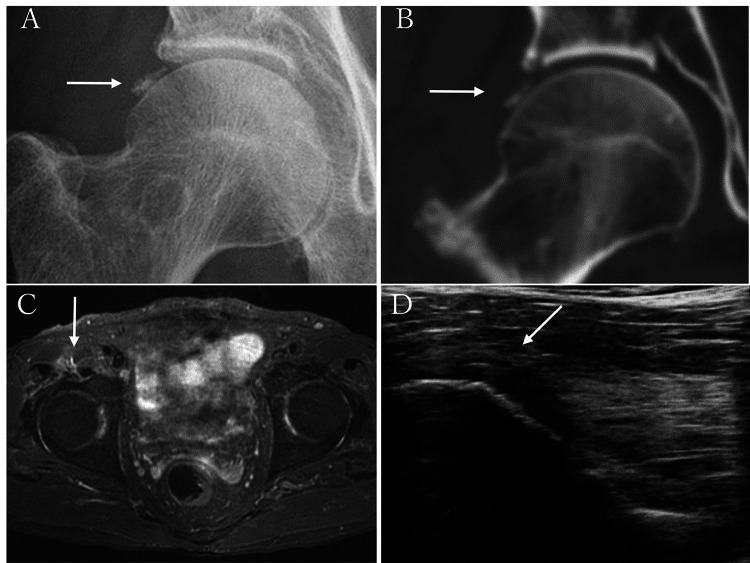
Radiographs, CT, MRI, and an ultrasound examination of the hip. Anteroposterior radiograph of the right hip showing calcification near the superior lip of the acetabulum (arrow, A). Computed tomography of the right hip showing calcification in the reflected head of the rectus femoris (arrow, B). Magnetic resonance imaging coronal T2-weighted fat-suppressed image. Magnetic resonance imaging showed a signal change in the reflected head of the rectus femoris with an inflammatory change of the peripheral soft tissue (arrow, C). Ultrasound longitudinal static scanning showed a calcification with an inflammatory change of the peripheral soft tissue (arrow, D).

Representative case (case 2): A 76-year-old male visited our hospital with a 3-day history of an acute onset of severe right hip pain and inability to bear weight. There was a tenderness point at the right hip. Pain limited all active movements of the hip. Radiographs, CT, MRI and an ultrasound examination of hip showed calcification outside the joint, suggesting calcific tendinitis of the rectus femoris (Figure 1A, 1B, 1C, 1D). 

**Figure 2:**
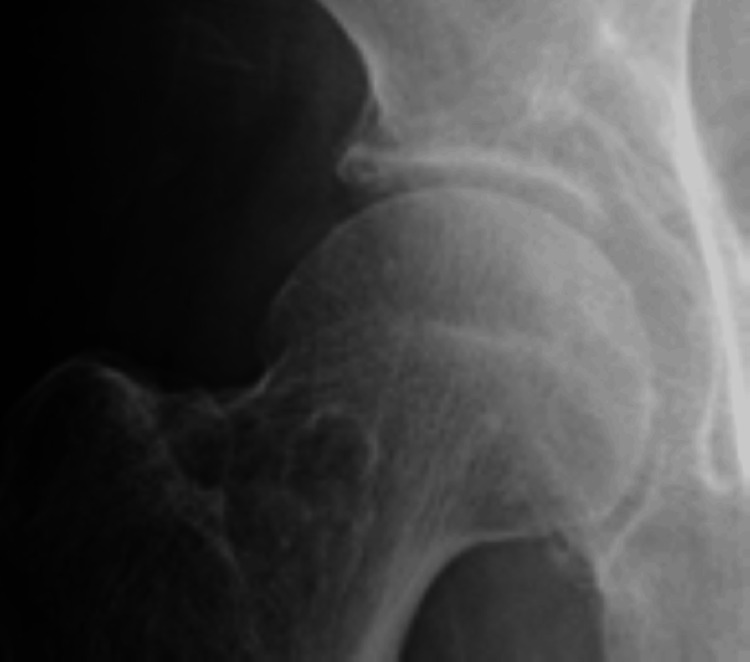
Anteroposterior radiograph of the right hip after 1 month of administration demonstrated disappearance of the calcification.

Oral NSAIDs and cimetidine were administered. Pain gradually decreased. 1 month later, he achieved a pain-free status and a radiographic examination showed the disappearance of calcification (Figure 2). 

**Figure 3:**
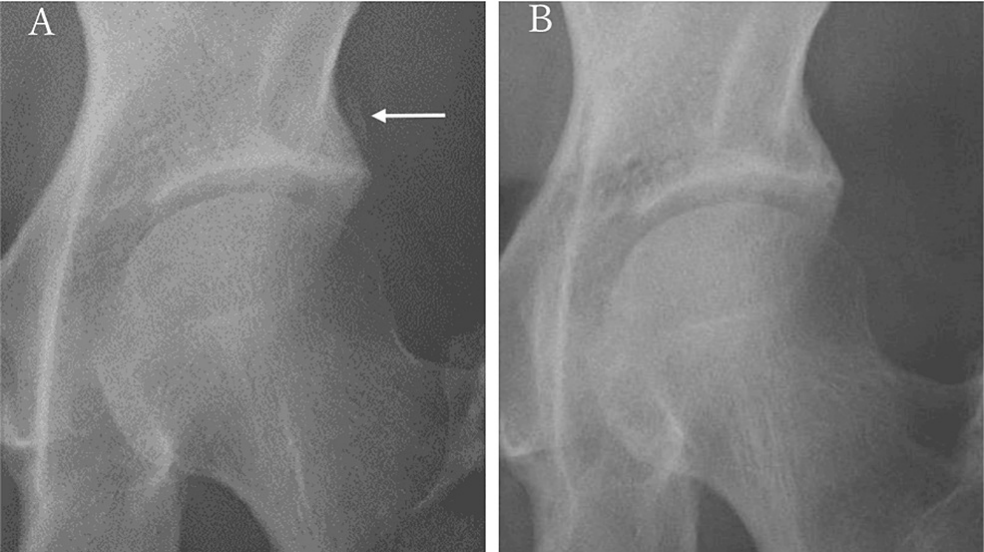
Radiograph of the left hip. Anteroposterior radiograph of the left hip showing calcification adjacent to the anterior inferior iliac spine (arrow A). Anteroposterior radiograph of the left hip after 1 month of administration demonstrated disappearance of the calcification (arrow B).

Representative case (case 5): A 44-year-old female presented with left hip pain. Radiographs of the hip showed calcification adjacent to the anterior inferior iliac spine (Figure 3A). Oral NSAIDs and cimetidine were administered. 1 month later, she achieved a pain-free status and a radiographic examination showed the disappearance of calcification (Figure 3B).

There were no symptoms or the recurrence of calcification in the 1-year follow-up.

## Discussion

Five patients (100%) achieved a pain-free status following the administration of NSAIDs and cimetidine, while three (60%) showed the complete disappearance of calcium deposits.

Calcific tendinitis involves the deposition of calcium hydroxyapatite crystals in the soft tissue and affects the joint. It most commonly involves the shoulder [1]. Calcific tendinitis around the hip joint is uncommon, and it commonly involves the greater trochanter [11]. Calcific tendinitis of the rectus femoris is relatively rare. The condition is usually monoarticular. Calcium deposits are located in tendons and soft tissues [12]. A previous study divided calcific tendinitis into acute, chronic, and latent forms [12]. The acute form is characterized by pain, local swelling, tenderness, restricted motility, and sometimes fever and is a self-limiting condition that generally resolves spontaneously within 4 weeks. However, a misdiagnosis is common, which delays treatment and recovery [7]. Calcific tendinitis is considered to be a reactive calcification process, and the process of calcific tendinitis is divided into four stages: precalcific, calcific, resorptive, and reparative. The degeneration of tendon substances into fibrocartilage and subsequent calcification is followed by vascular proliferation. In the resorptive phase, inflammatory findings were elicited because of calcium resorption. Patients may present with pain due to an increase in intratendinous pressure. In the reparative phase, a tendon may regain its original architecture [13]. Although definitive pathophysiological factors have not yet been identified, traumatic, genetic, and metabolic factors have been proposed as etiologies [14].

Treatment options include the administration of NSAIDs and cimetidine, shock wave therapy, local steroid injection, multiple puncture, or surgical interventions. The use of cimetidine is considered to be safer than other treatments.

Cimetidine is a histamine H2-receptor antagonist. A previous study on patients with hyperparathyroidism reported the normalization of parathyroid hormone and blood calcium levels following treatment with cimetidine [15]. Cimetidine was used to decrease parathyroid hormone levels in patients with secondary hyperparathyroidism. Although the underlying mechanisms currently remain unclear, cimetidine may affect other pathological conditions of heterotopic calcification [16]. Previous studies reported that cimetidine exhibited anti-inflammatory activity in rats by reducing nitro-oxidative stress. Cimetidine reduced interleukin-6 as well as matrix metalloproteinase-1 and -9 immunoexpression in rats [17].

A previous study demonstrated that the use of cimetidine was effective for calcific tendinitis of the shoulder [9]; 10 out of 16 patients achieved a pain-free status following the oral administration of cimetidine, while the disappearance of calcium deposits was observed in nine, reductions in four, and no change in three. Another study demonstrated that cimetidine was effective for calcific tendinitis of the elbow [10]; a pain-free status and the disappearance of calcium deposits were observed in all patients orally administered cimetidine. In this study, no pain occurred in any patient. The disappearance of calcium deposits was observed in three patients and reductions in two. These results suggest that cimetidine can be a choice for the treatment of calcific tendinitis of the rectus femoris. A previous study demonstrated that the use of famotidine which also included an H2-receptor antagonist, inhibited the expression of ossification markers in tendon cells under osteogenic conditions, and the oral administration of famotidine suppressed tendon calcification in mice [18].

Limitations

There are several limitations to this report. First, we did not have a control group. We could not exclude the possibility that calcifications reduced spontaneously. Second, we had a small number of cases. Third, we performed a short follow-up. Further study is required to elucidate the long-term effect of cimetidine.

## Conclusions

We investigated the effect of the administration of cimetidine for calcific tendinitis of the rectus femoris. It appears to be an effective treatment for this condition; however, the underlying mechanisms of action of cimetidine on calcific tendinitis have not yet been elucidated in detail. The results of this report warrant further controlled studies to investigate the effect of the administration of cimetidine.
